# Land Invasion by the Mudskipper, *Periophthalmodon septemradiatus*, in Fresh and Saline Waters of the Mekong River

**DOI:** 10.1038/s41598-019-50799-5

**Published:** 2019-10-02

**Authors:** Hieu Van Mai, Loi Xuan Tran, Quang Minh Dinh, Dinh Dac Tran, Mizuri Murata, Haruka Sagara, Akinori Yamada, Kotaro Shirai, Atsushi Ishimatsu

**Affiliations:** 1 Graduate School of Fisheries and Environmental Sciences, Nagasaki University, 1-14 Bunkyo-machi, Nagasaki, 852-8521 Japan; 2 School of Education, Can Tho University, 3/2 Street, Ninh Kieu District, Can Tho City Vietnam; 3 College of Aquaculture and Fisheries, Can Tho University, 3/2 Street, Ninh Kieu District, Can Tho City Vietnam; 4 Institute for East China Sea Research, Organization for Marine Science and Technology, Nagasaki University, 1551-7 Tairamachi, Nagasaki, 851-2213 Japan; 5 Atmosphere and Ocean Research Institute, The University of Tokyo, 5-1-5 Kashiwanoha, Kashiwa-shi, Chiba 277-8564 Japan

**Keywords:** Ecophysiology, Evolutionary ecology, Animal behaviour, Ichthyology

## Abstract

There has been a long-standing controversy about whether vertebrates emerged in the Paleozoic from marine or freshwater environments. Several hypotheses have proposed coastal, estuarine and riparian areas as sites of the transition. Here, we report the ecology of an amphibious fish *Periophthalmodon septemradiatus*, which we presume is in the process of niche expansion into terrestrial habitats from estuarine to freshwater environments along the Mekong River, Vietnam. Adult fish are highly terrestrial and have not been observed to venture into water during our survey. Courtship behaviour was observed, and fertilised eggs were recovered from burrows in both brackish and freshwater environments. The smallest fish collected at 12, 96, and 148 km from the river mouth were juveniles shortly after starting an amphibious life. These findings suggest reproduction in both brackish and freshwater environments. In contrast, otolith Sr:Ca ratio indicates larval hatching only in brackish water. Analysis of a 940-base pair (bp) segment of the mitochondrial cytochrome c oxidase subunit II and a 934-bp segment of the mitochondrial D-loop demonstrated no genetic segregation between populations. The fish may provide a unique opportunity to study how ambient salinity affects the biology and ecology of a living vertebrate during transition from water to land.

## Introduction

Emergence of vertebrates from water to land, which is thought to have occurred in the mid to late Devonian Period (approximately 390 to 360 million years ago), represents one of the most pivotal events in the history of life on Earth^1^. Discoveries of new fossils of transitional animals, e.g. elpistostegalian fishes (the fishes most closely related to tetrapods) and early tetrapods, during the last few decades have refined our knowledge on the process considerably, especially on how body structure changed accompanying the habitat transition^2–5^. These palaeontological studies have also given important clues to unveiling the environmental settings and ecosystem structures of the sites where the transition might have occurred^6^. The idea that vertebrates abandoned drying-up freshwater bodies in drought to seek larger remaining ponds^7^ had been widely accepted in the 20^th^ century, but has become questioned by more recent researchers^8^, particularly after the finding of trace fossils, which dated older than the first known body fossils of vertebrates, and was initially thought to indicate coastal transition to land^9^. In addition, the new analysis of stable isotopes of the bones of early tetrapods has lent support for the euryhalinity of these animals, which supposedly helped rapid global distribution and colonisation of different land masses^10^. Even though these palaeontological studies provide the most direct evidence for the process of land invasion by early vertebrates, the fossil records are inherently fragmentary, and some aspects of their ecology remain difficult to reconstitute from these materials. Some of these knowledge gaps may therefore be complemented by studying extant animals that are showing such a transition today^11^.

**Figure 1:**
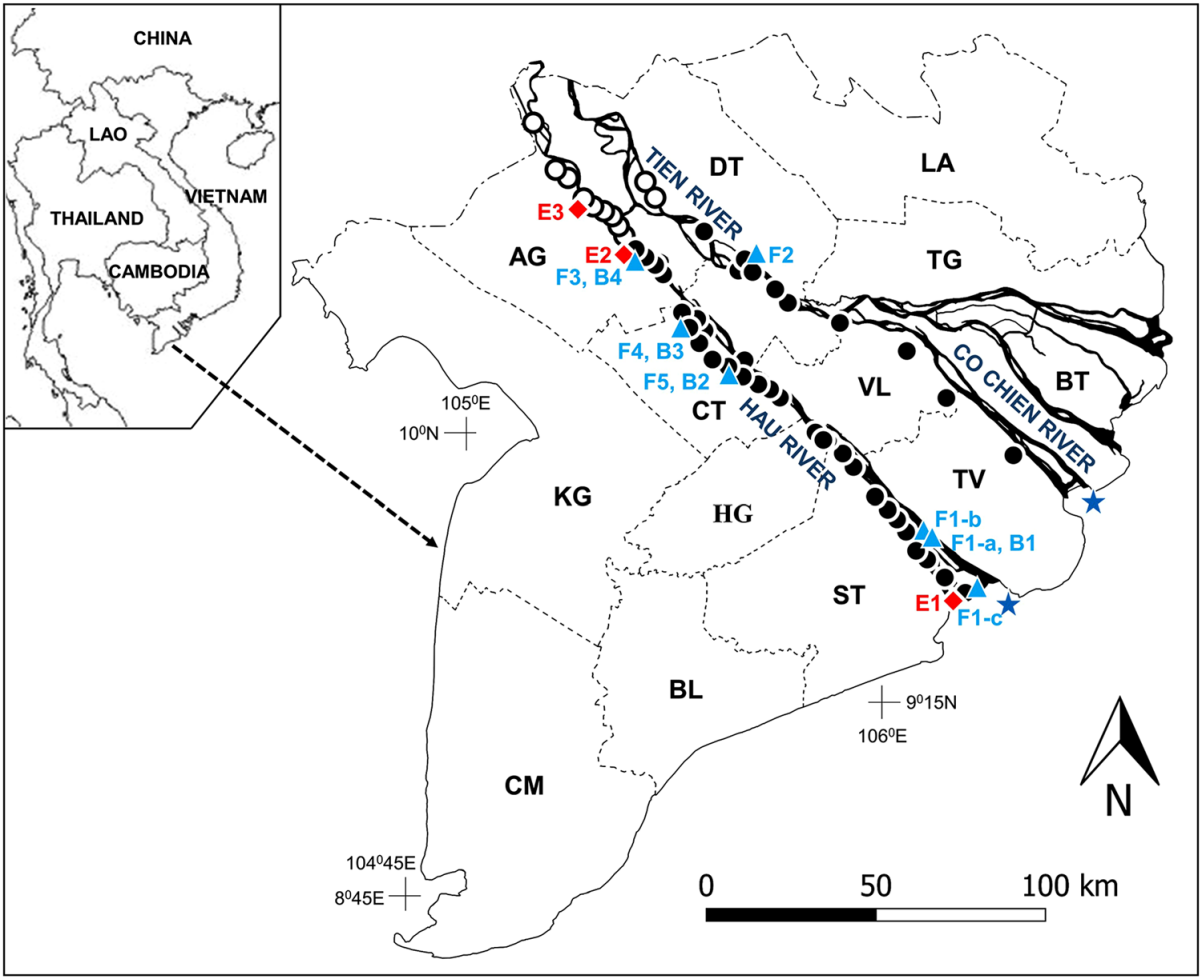
The distribution of *Periophthalmodon septemradiatus* along the two major channels of the Mekong River, the Hau River and the Tien River–Co Chien River. Filled circles represent sites where the mudskipper was observed, while open circles are where the mudskipper was not observed. The red diamonds labeled as E1 to E3 are the sites of environmental monitoring (see also Supplementary Table S1), and the blue triangles labeled as F-1 to F5 the sites of fish sampling (Supplementary Table S2). Burrow density was determined in close vicinity to four fish sampling sites (B1 to B4). Two stars represent the locations used to calculate the distances of each site from the river mouth. The abbreviations in the map represent the names of provinces or a city (CT). AG, An Giang; BL Bac Lieu; BT, Ben Tre; CM, Ca Mau; CT, Can Tho; DT, Dong Thap; HG, Hau Giang; KG, Kien Giang; LA, Long An; ST, Soc Trang; TG, Tien Giang; TV, Tra Vinh; VL, Vinh Long. The map was created with Qgis 3.4 (http://qgis.org/downloads/QGIS-OSGeo4W-3.4.7-1-Setup-x86_64.exe)^61^ and Microsoft Powerpoint - version 1904 (https://products.office.com/en-ie/powerpoint) for labels and icons.

Mudskippers are amphibious gobies belonging to the subfamily Oxudercinae^12^. These fishes usually inhabit intertidal mudflats of tropical and subtropical coasts and estuaries, and show various degrees of behavioural, morphological and physiological adaptations to terrestrial environment. During low tide, mudskippers emerge from water for various activities such as feeding and defending territories, while during high tide some species remain out of water but others retreat into burrows^13^. Courtship occurs only during low tide, and the mating pair enters a burrow while the mudflat is exposed. Eggs are laid on the wall of a spawning chamber within the burrow. Parental care comprises continuous addition of fresh air into the spawning chamber during low tides, and terminates with submersion of hatch-competent embryos during high tide^14^. Although goby lineages are estimated to have appeared in the latter half of the Eocene, approximately 48.7–36.2 million years ago^15^ (see also Reichenbacher *et al*.^16^ for the oldest goby fossil dating 19.1–20.4 million years ago) and thus are far more modern than the transitional animals of the Paleozoic, mudskippers could still provide insights into how land invasion would alter body structure and other life history traits. Despite the fact that most mudskippers are highly euryhaline^17,18^, their occurrence in low salinity waters has only been sporadically reported (see Discussion). Through our field survey in the Mekong Delta during the last four years, we found that one species of mudskippers, *Periophthalmodon septemradiatus*, inhabits over a 150 km stretch of the riparian zones along the Mekong River in Vietnam. The Mekong River flows as two main channels, the Hau River and the Tien River, through the Mekong Delta, and the Tien River further subdivides into the Co Chien River and a few other channels before reaching the coast (Fig. 1).

In this paper, we report on the basic ecology of *P. septemradiatus* inhabiting from brackish to freshwater reaches of the Mekong River, discuss how studying this species will benefit the understanding of vertebrate transition to land from waters of different salinities, and propose a possible scenario for the expansion of the distribution range of this species into freshwater reaches of the Mekong River.

## Results

### Fish distribution

*Periophthalmodon septemradiatus* was found up to 148 km from the river mouth of the Hau River in the An Giang Province and 155 km from the river mouth of the Co Chien River in the Dong Thap Province (Fig. 1). We found no individual of *P. septemradiatus* in the reaches further upstream from these spots to the border with Cambodia. The fish were abundant in tributaries, but were far fewer in the main channels; only small individuals were occasionally found around mangrove bushes in the banks of the main channel of the Hau River (no detailed survey was done along the Tien River–Co Chien River). In the lowermost sites (F1-a,b,c), *P. septemradiatus* occurred sympatrically with other mudskippers (*P. schlosseri, Periophthalmus chrysospilos, Ps. gracilis, Ps. variabilis*, and *Boleophthalmus boddarti*), but it was the only mudskipper species in the more upstream sites.

### Habitat environment

Semidiurnal fluctuation of water level was recorded in all the monitoring sites (including E3 where *P. septemradiatus* was not observed, 172 km from the river mouth, see Fig. 1 and Supplementary Table S1). The tidal range during spring tides at E1 (8 km from the river mouth) reached three metres while it was less than one metre at E2 and E3, which agrees with the data reported earlier^19^. Salinity measured at 20–30 cm above the river bottom remained zero at E2 and E3 irrespective of the season or time of the day, while it fluctuated with the tide at E1, ranging from zero to approximately 10 at high spring tides. Water temperature showed occasional daily fluctuations of <5 °C (25–30 °C) at E1, but it remained at about 30 °C at E2 and E3. The PO_2_ of river water ranged from 8.1 to 23.4 kPa (mean ± SD, 15.0 ± 6.5, N = 6). The habitats in tributaries were shaded by thick vegetation of Nipa palm and other plants, unlike the open mudflats in coastal areas where other species of mudskippers are abundant.

### Body size, morphometry and meristic characters

There was no significant difference in the relationship of standard length (SL, cm) and body mass (BM, g) between fish from the three sampling sites (F1-c, F4 and F5; Supplementary Table S2) as shown by comparison of residuals from the regression line for the pooled data (log BM = −1.876 + 3.077 × log SL, *r*^2^ = 0.962, H_2_ = 5.442, *P* = 0.066, Kruskal-Wallis one-way ANOVA, Supplementary Fig. S1). Morphometric analysis revealed statistically significant differences in the first dorsal fin base length (H_2_ = 9.694, P = 0.008), the second dorsal fin base length (F_2,27_ = 3.781, P = 0.036, one-way ANOVA), the caudal fin length (F_2,27_ = 8.181, P = 0.002), the pectoral fin length (F_2,27_ = 6.899, P = 0.004), the pectoral fin height (H_2_ = 8.871, P = 0.012), body width at the anus (H_2_ = 8.739, P = 0.013), head width (F_2,27_ = 10.860, P = 0.001), and eye diameter (F_2,27_ = 20.259, P = 0.001) between fish from F1-c, F4 and F5 (Supplementary Table S3). The number of fin rays was significantly different in the second dorsal (male only, H_2_ = 8.700, P = 0.013) and caudal fins (F_2,27_ = 4.870, P = 0.016) (Supplementary Table S4).

The smallest fish collected in F1-c (12 km from the river mouth), F5 (96 km) and F3 (148 km) were juveniles and already amphibious (total length 27, 24 and 25 mm for F1-c, F5 and F3, respectively). No larvae have yet been collected.

### Reproductive ecology

**Figure 2:**
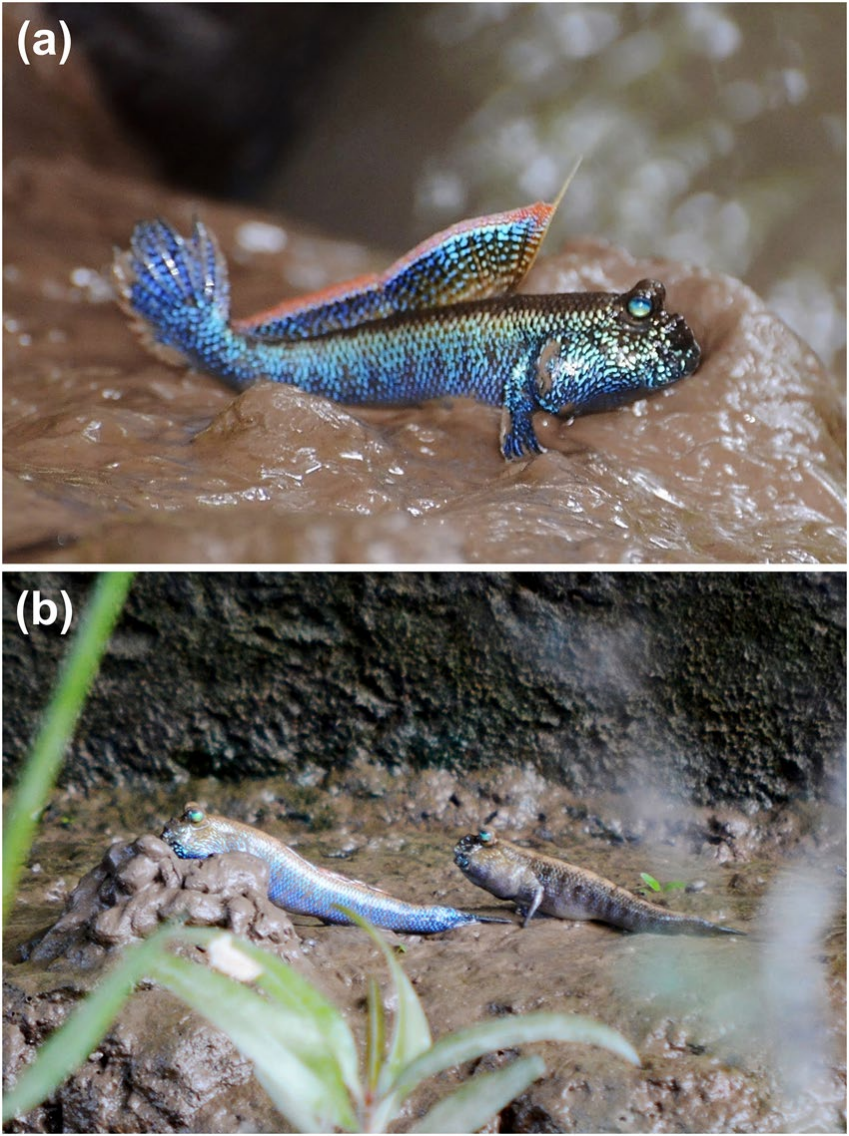
(**a**) A male of *Periophthalmodon septemradiatus* developing nuptial coloration. (**b**) A male *Periophthalmodon septemradiatus* about to enter his burrow followed by a female.

Courtship behaviour was observed throughout the distribution range. The reproductive behaviour seemed to be more frequent in the early rainy season (June) than in the other months, but we did not quantify it. Upon courtship, the male changed its body colour from a subdued brownish one to vivid metallic deep blue (Fig. 2a). Based on field observation, the body colour appears to be able to revert to the non-nuptial one within minutes. The female did not change body colour, and maintained a muted tannish colour even during courtship. A successful male then escorted the female to his burrow (Fig. 2b). The courtship was more frequent once the tide started to ebb at spring tide.

The burrow density was high along muddy banks of tributaries, but scarce along the main channel of the Hau River (Table 1, see also Fig. 1 and Supplementary Table S5) except in B1, where the burrow density was similar between the main channel and tributaries. The burrow density in the uppermost site (B4) was low even in tributaries. Eggs were collected at B1 (4 clutches), B2 (4) and B3 (2). Eggs were laid on the upper wall of the spawning chamber in a single layer. Mean (±SD) clutch size was 6600 ± 1600 (2 egg clutches each from the three sites), and there appears to be no trend for clutch size to vary with sampling sites. Eggs were elliptical, measuring 0.7–0.8 mm in long-axis and 0.5 mm in short-axis lengths with a clump of adhesive filaments at one end of the long axis. There was a small significant difference in the long-axis length and short-axis length between the sites (<10% of each axis length, one-way ANOVA), but we tentatively regarded these to be biologically insignificant due to the small sample size. Not all egg clutches could be used for clutch size estimation because complete recovery of eggs was not achieved in some cases.

**Table 1:** Burrow density (number m^−1^) along the main channel or tributaries of the Hau River.

Site ID	District, Province	Main channel	Tributary
B1	Cu Lao Dung, Soc Trang	0.11, 0.42, 0.65	0.32, 0.38, 0.50, 0.82
B2	Binh Thuy, Can Tho	0.00, 0.00, 0.04	1.11, 1.76, 1.79
B3	Thot Not, Can Tho	0.00, 0.07	1.05, 1.10, 1.64
B4	Chau Thanh, An Giang	0.00, 0.00, 0.07	0.13, 0.18, 0.44

The spawning chamber was filled with air (51.0 ± 3.7 ml/burrow, N = 3) as in other mudskippers^20^. Oxygen-rich conditions inside the chamber were also supported by the higher redox potential of the upper wall mud of the chamber (144 ± 48 mV, N = 6) than the values of the surrounding mud (−144 ± 46 mV, N = 6, P = 0.00004, df = 5, paired t-test). Burrow water showed the mean PO_2_ of 5.2 ± 1.9 kPa (N = 11) and salinity <0.3 except at B1 where no data were available.

**Figure 3:**
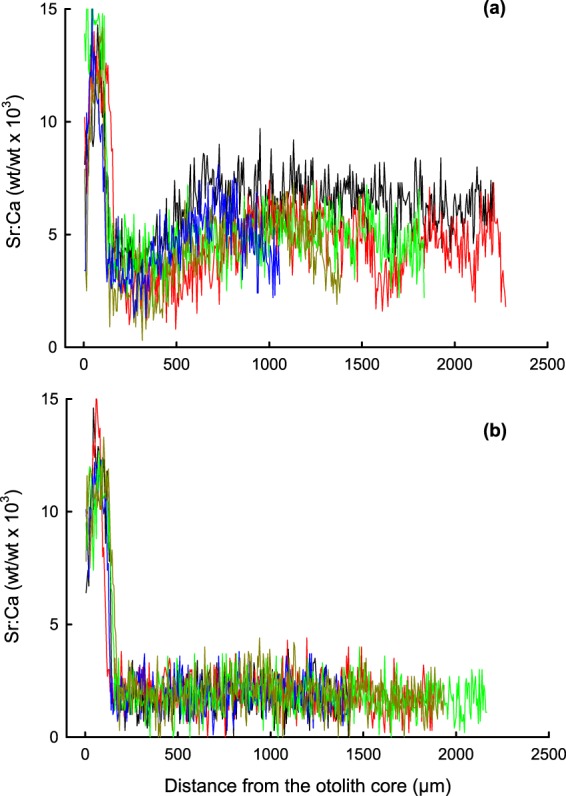
Sr:Ca ratio (weight/weight × 10^3^) of the sagittal otolith of *Periophthalmodon septemradiatus*. (**a**) The specimens were collected at F1-a, a brackish water site (N = 5). (**b**) The specimens were collected at F4, a freshwater site (N = 5). Different colours indicate different individuals.

Sr:Ca ratio of the sagittal otolith showed the mean values of 6.9–8.8 in the innermost “Core” segment, 11.4–12.2 in the “Peak” segment, and 1.9–4.8 in the outer “Periphery” segment (Table 2 and Fig. 3). There were significant differences in the Sr:Ca ratio in the “Periphery” segment between sites (H_4_ = 18.116, P = 0.001, Kruskal-Wallis one-way ANOVA), with the values from F1, F2 and F3 significantly higher than from F4 and F5. However, there is no correlation between the “Periphery” values and the distance from the river mouth. No significant difference was found for Sr:Ca ratio of “Peak” segments between five sites (H_4_ = 2.369, P = 0.668, Kruskal-Wallis one-way ANOVA). The Sr:Ca ratio at the innermost measurement point varied from 6.4 to 13.9 with one exceptional fish showing 3.4 (F1). The shift from the “Peak” to the “Periphery” segment always occurred within 200 μm from the otolith core (Fig. 3). Burrow water ion concentrations in the upper reaches of the Hau River (B2–4) were significantly higher than in adjacent river water for Na (H_2_ = 14.506, P = 0.001, Kruskal-Wallis one-way ANOVA), K (H_2_ = 21.177, P = 0.001), Ca (H_2_ = 20.710, P = 0.001) and Mg (H_2_ = 20.938, P = 0.001), but no difference was detected for Sr (H_2_ = 3.432, P = 0.180, Supplementary Table S6). No difference was detected between burrow and river water for any ion collected at B1.

**Table 2:** Sr:Ca ratio (weight/weight × 10^3^) of the sagittal otolith of *Periophthalmodon septemradiatus* collected at five sites along the Hau River (F1, F3–F5) and the Tien River (F2).

Site ID	Core segment	Peak segment	Periphery segment	Distance from the river mouth (km)
F1	6.9 ± 2.5 (4)	12.2 ± 0.99^a^	**4.80 **±** 0.81**^**a**^	27
F2	8.0 ± 0.3 (4)	11.8 ± 0.78^a^	**2.14 **±** 0.55**^**a**^	127
F3	8.5 ± 0.7 (3)	11.7 ± 0.61^a^	**2.16 **±** 0.13**^**a**^	148
F4	8.8 ± 0.9 (5)	11.4 ± 0.62^a^	**1.94 **±** 0.89**^**b**^	119
F5	8.8 ± 0.5 (4)	11.8 ± 0.27^a^	**1.94 **±** 0.18**^**b**^	96

Values are based on the data from five fish for each site except “Core segment” for which the number of fish are given in parentheses. Mean ± SD. The data with different letters in respective columns are significantly different (see text). No statistical test was applied to the data “Core segment” because of the low number of available data. The values of F1 were obtained from the sites F1-a and F1-b.

### Population structure

**Figure 4:**
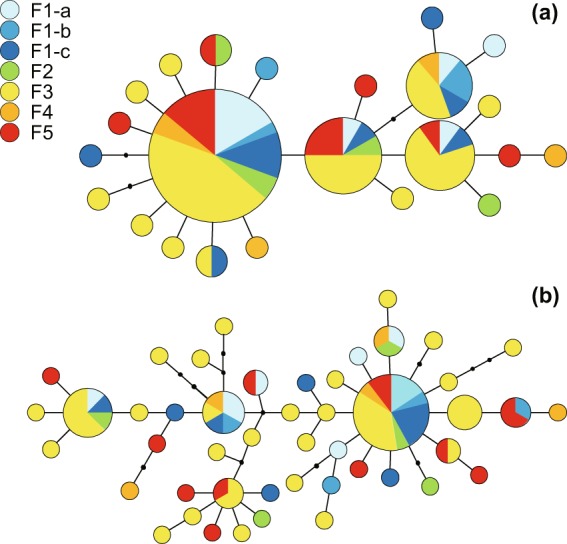
Maximum-likelihood haplotype network for cytochrome c oxidase subunit II (**a**) and D-loop (**b**). Each circle represents one haplotype, the size of circle corresponds to the abundance of individuals and the color indicates the sampling site (see Fig. 1 and Supplementary Table S2). Black dots indicate unsampled mutations (haplotypes).

For 679-bp COII, a total of 88 sequences were obtained from *P. septemradiatus*, revealing 24 polymorphic sites and 23 haplotypes (Supplementary Table S7). Overall haplotype diversity was H*d* = 0.796 and nucleotide diversity *π* = 0.00258. Likewise, for 828-bp DL, a total of 88 sequences were obtained, revealing 34 polymorphic sites and 47 haplotypes (Supplementary Table S8). Overall haplotype diversity was H*d* = 0.940 and nucleotide diversity *π* = 0.00471. Haplotype richness (H*r*), which was calculated with a sample size of 4, was correlated with haplotype diversity values (Spearman correlation coefficient: *r* = 0.935 for COII, *r* = 1.000 for DL, P < 0.05 in both cases). There were, however, no clear patterns of genetic diversity across the sampling sites. The most common haplotype of each gene was observed in all sampling sites (Fig. 4).

Population pairwise ɸst values showed no genetic differentiation between any pair of the sampling sites using the standard *p* < 0.05 as a significance level (Supplementary Table S8). No isolation by distance was also observed (*r*^2^ = 0.018, *p* = 0.743 for COII, *r*^2^ = 0.053, *p* = 0.088 for DL). GenBank/EMBL accession numbers are from LC421229 to LC421404.

## Discussion

This is the first finding of an amphibious fish inhabiting and breeding over a range from a saline environment of an estuarine island to completely freshwater habitats of the same river. *Periophthalmodon septemradiatus* colonises the riparian zone along the two major channels of the Mekong River (Fig. 1), feeding and courting (Fig. 2) out of water on the bank of tributaries, and spawning eggs in burrows excavated in the bank. Although mudskippers are known to be generally euryhaline^17,18^, only two species (*P. septemradiatus* and *Periophthalmus weberi*) have been recorded from freshwater environments. According to the database of the fish distribution in the Southeast Asia, *P. septemradiatus* was previously collected at 90 and 150 km upstream from the river mouths of the Hau River and the Co Chien River, respectively (Supplementary Fig. S2)^21^. Brief description of each sampling site is given in the database, but no ecological data are available. In Malaysia, *P. septemradiatus* was found in the Selangor River in the Malay Peninsula, about 15 km from the river mouth, where salinity was 0 at low tide and 1–3 during extreme high tides^22^. *Periophthalmus weberi* was collected at a creek in the Northern Territory, Australia, approximately 60 km upstream from the coast (salinity 0.1)^23^. More extremely, *Ps. weberi* was found at about 250 km from the river mouth of the Fly River, Papua New Guinea^24,25^. Unfortunately, the ecology of *Ps. weberi* has not been investigated to date. Other examples of euryhaline, amphibious teleosts include mangrove killifish *Kryptolebias marmoratus*, climbing perch *Anabas testudineus*, and swamp eels *Monopterus* spp. However, the habitat conditions, ecology, and reproductive biology of these fishes are scarcely known^26^. Thus, further investigation of *P. septemradiatus* could offer a unique window through which to glimpse how environmental salinity affects the biology and ecology of an animal during the process of niche expansion from aquatic environment, and would thereby complement our understanding about where early transitional vertebrates emerged from water in the Paleozoic.

*Periophthalmodon septemradiatus* is probably one of the most terrestrial fishes living today. During four years of our field survey in the Mekong Delta, we did not see *P. septemradiatus* ventures into water. The high terrestriality of *P. septemradiatus* is further supported by the finding that ants, *Dolichoderus* sp., constituted a substantial portion of the total gut content (>80% by biovolume analysis)^27^. The *Dolichoderus* ants scavenge on the ground floor but also forage for insect secretions on low vegetation and trees^28^. Along the banks where *P. septemradiatus* occurs, the ants rarely intrude onto the soft mud substrate of the intertidal zone, but occur in areas above it. These observations indicate that *P. septemradiatus* feeds largely, if not mostly, in areas beyond the water’s edge. Another example of a highly terrestrial mudskipper is *Ps. minutus* inhabiting the highest intertidal zone in Australia. The fish is able to withstand continual emersion of more than 20 days during some neap tides by retreating into its burrow^29^. *Ps. minutus* also ingests ants in addition to crabs and other animals^29^. Carnivory is presumably a crucial factor that allows amphibious fishes to extend habitat usage and exploit available food resources. In contrast, herbivorous amphibious fishes such as *Boleophthalmus* mudskippers^30^ and several rockskippers (Blenniidae)^31^ are trophically reliant on the lower intertidal zone because of high moisture conditions necessary for the growth of micro- and macro-algae that these fishes feed on. Early vertebrates are believed to have been all carnivorous^1^.

None of the environmental parameters measured in this study seems to be limiting the fish’s distribution range. In this regard, it is interesting to note that marine/brackish species of live bivalves were collected to 160 km from the river mouth of the Tien River–Co Chien River^19^. The most upstream sites of fish occurrence were nearly at the same distance from the river mouth both in the Hau River and in the Tien River, suggesting a common limiting factor for their distribution. The fish’s near absence along the main channels is probably due to instability of the environment, where mud banks are subject to frequent erosion and deposition. Mangrove bushes probably stabilise local bank conditions, create local food webs and thereby provide a suitable habitat for *P. septemradiatus*.

It should also be noted that *P. septemradiatus* does not usually inhabit coastal tidal flats. In contrast, most other oxudercine gobies in the Mekong Delta almost exclusively inhabit coastal zones (Supplementary Fig. S2). There are records of inland collection of oxudercine gobies in the Ca Mau Province^21^. However, there are extensive canal systems and thick mangrove forests in a large part of the Ca Mau Province, and the salinity in these systems can be 40–45 in the dry season and 8 in the rainy season^32,33^. Thus, the inland records of mudskipper occurrence in the Ca Mau Province were likely due to fish migration into those brackish/seawater canal systems. *Parapocryptes serperaster*, a cryptic oxudercine goby, was collected up to 85 km from the river mouth of the Hau River^21^. The reason(s) for the absence of *P. septemradiatus* in the coastal mudflats is currently unknown, but the possibility includes their intolerance to seawater at some life stage(s) or competition with other mudskippers and intertidal animals.

Courtship display was observed throughout the distribution range of *P. septemradiatus*. Moreover, the fertilised eggs were collected from burrows in both fresh (B2 and B3, Fig. 1) and brackish water (B1) habitats. These indicate larval hatching in both fresh and brackish water environments. On the contrary, the otolith Sr:Ca data from all five sites indicate larval hatching only in saline conditions, and subsequent migration to even higher salinity waters before settling in brackish water at F1 or freshwater at the other sites (Table 2 and Fig. 3). Environmental salinity is usually regarded as the most robust determinant of otolith Sr:Ca ratio^34,35^, although the ratio can also be influenced by factors other than salinity, e.g., temperature, ontogeny, and species^36^. The high Sr:Ca ratio was not due to lower Ca content in the otolith primordial region, the mass % of CaO in the otolith ranging 50 to 55 throughout the transect from the core to the edge of the otolith.

Two hypotheses may be proposed to elucidate the recruitment of larval *P. septemradiatus* to its freshwater habitats. First, larvae hatch in freshwater and migrate downstream to brackish/marine waters and migrate back to the upstream reaches, and the high Sr:Ca ratio in the otolith is due to physiological status or other factors, and does not reflect environmental salinity^37^. Second, larvae that hatch in freshwater are abortive and those that hatch in brackish/marine waters recruit to the entire distribution range and thus constitute the exclusive source of all the populations. The Sr concentration of burrow water was not significantly higher than the adjacent river water (Supplementary Table S6), precluding the possibility of local accumulation of the element in burrows. None of the DNA analyses indicated segregation of fish populations in our samples, though the analyses may imply only the absence of distinct segregation that could be detected by mitochondrial markers. More field studies should be conducted to reveal larval occurrence in fluvial and coastal waters. In addition, larval rearing needs to be done to examine the relationship between Sr and Ca concentrations in ambient water and otolith Sr:Ca ratio; the growth rate at ambient temperatures in the Mekong River region; and salinity tolerance of larvae and juveniles under controlled conditions. Recently, Dinh *et al*.^38^ studied monthly changes in gonadal development of *P. septemradiatus*, and inferred that the fish was iteroparous.

Irrespective of routes of its recruitment, *P. septemradiatus* is most likely emerging from both brackish and fresh water, as suggested by the occurrence of juveniles with the total length of 24 to 27 mm in the three collection sites. The wide salinity range in which *P. septemradiatus* emerges provides an opportunity to study whether calcium concentration in environmental water affects skeletal development of animals in transition from water to land. Fishes take up calcium, a major constituent of fish bones, mostly from water through the gills^39^. However, the gills invariably diminish and atrophy with increasing dependence on aerial respiration both in extant fishes^40^ and in Devonian transitional animals^1^. It seems therefore possible that the difference in calcium concentration in ambient water affects skeletal modification during the mudskipper’s metamorphosis from larvae (pelagic) to juveniles (amphibious)^41^. Our preliminary examination demonstrated a number of subtle, but significant, differences in body morphologies between sites (Supplementary Tables S3 and S4), indicating a need for further detailed study on the skeletal anatomy. A recent study demonstrated that juveniles of *Polypterus senegalus* reared in shallow water (depth 3 mm) for 8 months developed altered anatomy of the pectoral girdles as compared with water-reared control fish, which resembles the ancient anatomical changes in stem tetrapods during water-to-land transition^42^.

Nguyen *et al*.^43^ investigated the coastal evolution of the Mekong Delta. According to their analysis, the coastline ran near the present-day border between Vietnam and Cambodia 6000–5000 years ago when the sea level was approximately 5 m higher than today^44^, and since then migrated rapidly to form the present-day Mekong Delta. The coastline was located at the upper-stream periphery of the *P. septemradiatus* distribution range approximately 4500 years ago. Given these data, we hypothesise that *P. septemradiatus* initially inhabited the brackish-water zone of the then Mekong Delta, and with the rapid progradation of the delta, it expanded the habitat into freshwater reaches by remaining where they were left behind by the progression of the coastline, as a consequence of the high degree of freshwater tolerance and the capacity to exploit terrestrial animal food resources. Other mudskippers, both herbivorous and carnivorous, may have been precluded from the area that became increasingly distant from the coast, because of dwindling availability of food resources for them due to general desalination of the surroundings and environmental instability as discussed earlier. Annual flooding with the peak water levels 3–4 m above usual as measured near the upper distribution limit of *P. septemradiatus*^19^ must have added to the instability of the local riparian ecosystem structure. Thus, both herbivorous and carnivorous mudskippers would not be able to secure stable, sufficient food resources at the river water’s edge, unless they evolved a high degree of freshwater tolerance and terrestriality, enabling them to venture onto land in search for novel food resources. Extrapolating this line of reasoning, it can be speculated that some of euryhaline, transitional vertebrates of the Paleozoic era initially occurred in coastal waters, evolved the capacity of aerial respiration and terrestriality, and expanded their distribution to freshwater reaches of a large river through progradation of deltaic regions, as we hypothesise for *P. septemradiatus*. A broad range of habitats are advocated as possible sites of the evolution from early sarcopterygian fishes to tetrapods in the Devonian, including estuaries, marginal to fully marine, river channels, flood deposits, tidal pools in a deltaic region, and flooded woodland^45–48^. The hypothesis needs to be tested through more extensive field surveys coupled with statistical biogeography, more detailed genetic analyses including molecular nuclear markers, and laboratory physiological experiments using fishes of different developmental stages.

## Methods

### Study sites

Field study of *Periophthalmodon septemradiatus* was carried out mainly along one main channel of the Vietnamese part of the Mekong River, the Hau River, for the period of December 2015 through June 2018. In addition, we also conducted a field survey along another main channel, the Tien River–Co Chien River, in five trips to obtain complementary data. In total, 10 field trips were made (see Supplementary Table S9). In the December 2015 survey, we confirmed fish occurrence at two sites along the Hau River in the Vinh Long Province (81 and 87 km from the river mouth) and one site along the Tien River in the Dong Thap Province (98 km). During the 9 subsequent field trips, we continued to study fish occurrence along the Hau River and the Tien–Co Chien River. Sampling sites were selected on the basis of fish abundance, surrounding environmental conditions (thickness of vegetation, traffic, pollution), and safety. The sites of data logger installation were determined on the basis of easy access and safety. Because of practical limitations imposed on conducting field surveys in the country and rapidly changing environmental conditions of the Mekong Delta, it was difficult to sample fish and monitor environmental conditions in a more systematic way.

### Environmental measurements

Water depth, temperature and salinity of river water were monitored using data loggers; HOBO U20L for water depth and temperature and HOBO U24-001 and −002 for water conductivity (Onset Computer Corp. Massachusetts, USA) at three sites including the lower and upper limits of *P. septemradiatus* distribution (E1 and E2, see Fig. 1 and Supplementary Table S1), and further up where no specimens of *P. septemradiatus* were found (E3, see Fig. 1 and Supplementary Table S1). Water conductivity was determined also with a probe (Horiba 9382, Tokyo, Japan) and a portable conductivity meter (Horiba ES-51, Tokyo, Japan), and used for correcting the data obtained by the conductivity loggers. Water conductivity was converted to salinity using PSS-1978^49^. Dissolved oxygen (DO) concentration of river water was determined with a probe (Horiba 9520) and a meter (Horiba D-55), which was calibrated every day with a sodium nitrite solution and humidified air. The obtained DO concentration was converted to oxygen partial pressure using measured water temperature, salinity and oxygen solubility coefficient^50^. At each site, the data loggers were set vertically, guarded in a perforated PVC pipe (diameter 9 cm, length 90 cm) which was capped on each side. The pipe was tied to a stick pushed into the bottom sediment to position the data loggers 20–30 cm above the river bottom. Another depth data logger was kept in air for compensation for changes in barometric pressure. Sampling frequency was set at 5 minutes and sampling duration was 5 days or longer. The data were extracted to Excel files according to the instruction manual.

During burrow density determination (see Reproductive ecology), river and burrow water samples were collected, immediately filtered (0.45 µm), acidified to pH 2 by the addition of concentrated nitric acid, and stored at room temperature until analysis. Na, K, Ca, and Mg concentrations of river and burrow water were analysed with an ICP-AES (ULTIMA2, Horiba, Tokyo). Sr concentration was determined with ICP-QQQ (Aglient 8800, Aglient Technologies, Tokyo).

### Observations of fish occurrence

Fish occurrence was surveyed during low tide along the mud banks of the main channel and tributaries of the Hau River and the main channel of the Tien River-Co Chien River. We used binoculars, digital cameras and video recorders to identify species, and recorded fish behaviours. We took special care before concluding the absence of *P. septemradiatus* in a specific site, by observing some hundred metres of nearby river banks.

We used the keys by Takita *et al*.^51^ for field identification of mudskippers. Accordingly, *P. septemradiatus* had a brown body, lighter on the dorsal surface, with a dark horizontal line, running dorso-laterally from the eye and posteriorly becoming a row of dark spots. Numerous small dark speckles occurred on the snout, opercles and the flanks. The young had white speckles (instead of dark ones) laterally on the trunk, and the dorso-lateral row of dark spots was obscured posteriorly. In addition, the eyes of *P. septemradiatus* often appeared blue-green in colour, which was used as an additional characteristic to confirm species identification in the field. For collected samples, we further confirmed the occurrence of two rows of teeth in the upper jaw, a key characteristic for distinguishing *Periophthalmodon* from *Periophthalmus*^52^.

### Fish sampling and measurements

In this study, a total of 284 individuals were collected. 111 fish were collected on the mud bank in December 2017 using a baited hook and line for a comparison of the relationships of body mass and standard length between sites (see Supplementary Table S2). These fish were anaesthetised by immersion in a solution of FA-100 (DS Pharma Animal Health, Osaka, Japan). After determination of body mass and standard length, the fish were allowed to recover in river water and released, except 10 individuals from each site, which were euthanised in a concentrated FA100 solution, preserved in 10% neutralised formalin, and used for the determinations of morphometric and meristic parameters. In addition, 4 fish from F3 (the upper limit of fish distribution in the Hau River) were collected for the determinations. We measured 16 morphometric and 10 meristic parameters reported in Jaafar *et al*.^53^. 88 fish were used for DNA analysis, of which 20 were also used for otolith Sr:Ca analysis. Fish for DNA analysis were collected by dip nets in 2016 and 2017 as shown in Supplementary Table S2. After collection, they were sacrificed by immersion in ice water, measured for total and standard lengths, and preserved in 96% ethanol. These fish were preserved in the laboratory for further morphological analyses. A total of 76 individuals collected on April 11 (F3, 35 fish), 12 (F5, 14 fish) and 14 (F1-c, 27 fish), 2017 were not used in this study, and also preserved in 96% ethanol in the laboratory. We did not sample fish in 2015 or 2018, because the 2015 survey was preliminary and the 2018 surveys focused on environmental analyses and reproductive ecology. Unequal sample size between sites was partly due to fish abundance at each site and the assumed importance of fish population at the upper-limit site (F3) for the study.

### Reproductive ecology

Density and physicochemical conditions of *P. septemradiatus* burrows were studied along the bank of the Hau River and its tributaries in June 2018 (see Supplementary Table S5). Transects of 15–30 m were set parallel to a stream (ca. 3 m from the water’s edge) and the locations of burrows along the transects were recorded. Burrows were initially located by the shape and size of the openings. They were then emptied of water and excavated to confirm its identity on the basis of configuration (two sloped shafts that led to a dome-shaped spawning chamber at the bottom, often with upper surface of the spawning chamber a bright brownish color, Quang Minh Dinh *et al*. in preparation). For several burrows at each transect, burrow water was analysed for DO concentration with a probe (Horiba 9520) and a meter (Horiba D-55). The DO probe was inserted directly through a burrow shaft into the standing water inside. Redox potential of the upper surface of the spawning chamber and surrounding mud was determined with a probe (Horiba 9300) and a meter (Horiba D-55), immediately after excavation. After the measurement of redox potential, the horizontal cross section of the chambers was photographed and measured for their dimensions. Burrow air was sampled by the method reported earlier^20^. When eggs were found, they were transferred onto a piece of sponge soaked in water, and brought back to the laboratory. Eggs were photographed with a stereoscope and a camera for determination of egg and clutch size. Several egg clutches were used for preliminary trials of incubation in different salinities. Courtship behavior was photographed in several trips.

To elucidate migration history of larvae and juveniles, we determined Sr:Ca ratio of the sagittal otolith (Supplementary Table S2). Fish were preserved in 96% ethanol and brought back to Can Tho University. The otoliths were excised under a dissecting microscope and packed for bringing them to Japan. The otoliths were processed with the method reported earlier^54^, and analysed for Sr and Ca concentrations in a line along the longest axis from the core to the edge by a wavelength-dispersive X-ray stereoscopy using electron microprobe analyzer (JXA-8230, JEOL) housed at the Atmosphere and Ocean Research Institute, The University of Tokyo. Microscopic observation of the otolith samples confirmed that the otolith core was properly exposed and that Sr and Ca analysis started from the core. CaSiO_3_ and SrTiO_3_ were used as quantitative standards. All Sr:Ca ratios were expressed as weight ratios. Fish from F1-a, and F1-b were lumped as one group because of their close vicinity to each other.

### DNA sequencing

Total DNA was extracted from approximately 30 mg of tissue (muscle or fin) using a standard DNA lysis solution containing Proteinase K. A 940-base pair (bp) segment of the mitochondrial cytochrome c oxidase subunit II (COII) and a 934-bp segment of the mitochondrial D-loop (DL) were PCR-amplified with the newly developed primers, Pn.sept_CoII_F1 (5′-ACACATTTGAAGAGCCTGC-3′) and Pn.sept_CoII_R1 (5′-AGCTTAAAAGGCTGACGC-3′) for COII, and Pn.sept_DL_F1 (5′-TAGCTCCCAAAGCTAGCATTC-3′) and Pn.sept_DL_R1 (5′-TCAGGACCAAGCTTTTGTGC-3′) for DL. The PCR amplification was carried out in a reaction volume of 25 μl containing 1X EmeraldAmp MAX PCR Master Mix (TaKaRa Bio Inc.), 400 nM of forward, and reverse primer, 1 μl of 10-fold diluted DNA, and the remaining volume made up by nuclease-free water, at the following conditions: initial denaturation at 94 °C for 1 min 30 sec, 30 cycles of amplification with each cycle containing 94 °C for 30 sec, 50 °C (COII) or 60 °C (DL) for 30 sec, 72 °C for 1 min 30 sec (COII) or 2 min (DL), and a final extension at 72 °C for 5 min. The amplicons were purified using ExoSAP-IT (Thermo Fisher Scientific Inc.), and sequenced by outsourcing (FASMAC Co., Ltd.).

### Data analysis

To compare body mass–standard length relationships of fish from the three sampling sites, the residuals from the regression of the pooled data were analysed by one-way ANOVA. Sr:Ca data along the longest axis of each otolith were binned into “Peak” segment where Sr:Ca ratios were >10, “Core” segment central to “Peak” with Sr:Ca ratio <10 (but in some fish there was no “Core” area), and “Periphery” segment peripheral to “Peak” with Sr:Ca ratio <10. Mean values were obtained for each segment of each fish, and grand mean values were calculated for each site. One-way ANOVA was applied to detect statistical difference between sites. One-way ANOVA was also used for the comparison of morphometric and meristic data and for the comparison of river and burrow water ion concentrations between sites. When normality test failed, non-parametric test was used (Kruskal-Wallis one way of ANOVA on ranks), followed by Tukey or Mann-Whitney test for pairwise multiple comparisons.

DNA sequences were aligned, edited and trimmed to a common length using MAFFT version 7^55^ and GENETYX 12.0.4 (GENETYX Corp.). The number of haplotypes (*h*), haplotype diversity (H*d*), nucleotide diversity (*π*) was calculated using DnaSP 5.10^56^. We used Contrib 1.02^57^ to obtain haplotype richness after rarefaction (rarefied allelic richness) by taking into account differences in sample size. Arlequin 3.5.2.2^58^ was used to calculate population pairwise ɸst (an analogue of *F*st that includes sequence divergence) with 10,000 permutations. Isolation by distance was assessed by plotting pairwise ɸst against geographical distance (km) and using a Mantel Test with 10,000 permutations. To infer relationships between populations and sampling sites, a haplotype network for each gene was created using Haplotype Viewer^59^ (http://www.cibiv.at/~greg/haploviewer) with a Maximum-likelihood (T92 model for COII, T92 + G + I model for DL) tree reconstructed by MEGA 7.0.18^60^.

Values are expressed as means ± 1 standard deviation where possible.

This study was approved by the Animal Care and Use Committee of Institute for East China Sea Research, Nagasaki University and the Department of Research Affairs, Can Tho University. The methods used in this study were carried out in accordance with the guidelines of the stated committee and department.

## Supplementary information

Supplementary Information

## Data Availability

The datasets generated during and/or analysed during the current study are available from the corresponding author on reasonable request.
