# Supplementary material for: Land Invasion by the Mudskipper, Periophthalmodon septemradiatus, in Fresh and Saline Waters of the Mekong River
Source: Sci Rep. 2019 Oct 2;9:14227. doi: 10.1038/s41598-019-50799-5 (PMC6775124; doi:10.1038/s41598-019-50799-5)
Supplement: Supplementary file 1 — Supplementary Information [file 41598_2019_50799_MOESM1_ESM.pdf]

Land Invasion by the Mudskipper, *Periophthalmodon septemradiatus*, in Fresh and Saline Waters of the Mekong River

Hieu Van Mai, Loi Xuan Tran, Quang Minh Dinh, Dinh Dac Tran, Mizuri Murata, Haruka Sagara, Akinori Yamada, Kotaro Shirai and Atsushi Ishimatsu

Supplementary Information

Table S1. The locations of environmental monitoring along the Hau River, Mekong Delta, Vietnam

Site ID	District, Province	Latitude	Longitude	Distance from the river mouth (km)
E1	Cu Lao Dung, Soc Trang	9°30'35.22"N	106°13'43.02"E	8
E2	Chau Thanh, An Giang	10°26'57.78"N	105°22'20.76"E	150
E3	Chau Phu, An Giang	10°34'41.46"N	105°14'26.28"E	172

Environmental monitoring was conducted in April, September and December 2017 and in June, 2018

Table S2. Summary of fish sampling during this study along the Hau River, Mekong Delta, Vietnam

Sampling date	Site ID	District, Province	Latitude	Longitude	Nd (No)	Ns (Nm)	Distance from the river mouth (km)
Sept. 29, 2016	F1-a	Cu Lao Dung, Soc Trang	9°38'26.28"N	106°09'9.16"E	10 (2)		27
Sept. 30, 2016	F2	Cao Lanh, Dong Thap	10°24'39.96"N	105°43'10.62"E	5 (4)		127
Dec. 1, 2016	F3	Chau Thanh, An Giang	10°26'31.80"N	105°23'26.10"E	5 (3)		148
Dec. 5, 2016	F4	Thot Not, Can Tho	10°13'0.54"N	105°33'7.32"E	5 (5)		119
Dec. 8, 2016	F1-b	Cu Lao Dung, Soc Trang	9°39'31.58"N	106°9'38.20"E	4 (2)		27
Apr. 11, 2017	F3	Chau Thanh, An Giang	10°26'31.80"N	105°23'26.10"E	10 (0)		148
Apr. 12, 2017	F5	Binh Thuy, Can Tho	10° 2'58.62"N	105°43'25.50"E	13 (0)		96
Apr. 14, 2017	F1-c	Cu Lao Dung, Soc Trang	9°33'0.44"N	106°15'21.54"E	10 (0)		12
June 18, 2017	F3	Chau Thanh, An Giang	10°26'31.80"N	105°23'26.10"E	26 (0)		148
Oct. 6, 2017	F5	Binh Thuy, Can Tho	10°2'58.85"N	105°43'25.95"E	0 (4)		96
Dec. 15, 2017	F1-c	Cu Lao Dung, Soc Trang	9°33'0.44"N	106°15'21.54"E		45 (10)	12
Dec. 20, 2017	F3	Chau Thanh, An Giang	10°26'31.80"N	105°23'26.10"E		0 (4)	148
Dec. 21, 2017	F4	Thot Not, Can Tho	10°13'0.54"N	105°33'7.32"E		32 (10)	119
Dec. 23, 2017	F5	Binh Thuy, Can Tho	10° 2'58.62"N	105°43'25.50"E		34 (10)	96

Nd, the number of fish used for DNA analysis. No, the number of fish used for otolith analysis. All fish used for otolith analysis were also used for DNA analysis, except on Oct. 6, 2017 when the fish were used for otolith analysis only. Ns, the number of fish used for body mass and standard length determinations. They were released after the determinations, except for those used for morphometric analysis (Nm). On Dec. 20, 2017, fish were collected exclusively for morphometric analysis. Fish were not sampled in trips in December 2015, June 2016, or January and June 2018.

Table S3. Summary of morphometric analysis of *Periophthalmodon septemradiatus* collected from four sites along the Hau River

	F1-c	F5	F4	F3
% of Standard length				
Head length	29.6 ± 0.55	29.5 ± 0.89	30.2 ± 1.68	30.1 ± 0.54
Predorsal length	38.2 ± 1.28	38.1 ± 1.17	38.0 ± 1.56	38.6 ± 0.26
**First dorsal fin base length**	**16.2 ± 0.65[a] (3.6,4.5,5.7)**	**15.4 ± 1.77[ab] (7.7)**	**13.4 ± 2.53[b]**	14.4,16.0 (4.7,5.2)
**Second dorsal fin base length**	**24.9 ±1.21[a]**	**23.0 ± 1.65[b]**	**23.9 ± 1.74[ab]**	24.6 ± 1.95
Anal fin length	17.7 ± 0.99	18.0 ± 1.22	17.3 ± 1.19	17.2 ± 0.62
Pelvic fin base length	6.3 ± 0.56	6.4 ± 0.37	6.2 ± 0.43	6.4 ± 0.32
**Caudal fin length**	**25.7 ± 0.85[a]**	**26.0 ± 1.20[a]**	**24.3 ± 0.95[b]**	27.0 ± 1.90
**Pectoral fin length**	**19.8 ± 0.94[ab]**	**20.7 ± 1.20[a]**	**18.8 ± 1.20[b]**	21.5 ± 0.64
**Pectoral fin height**	**8.3 ± 0.28[a]**	**8.1 ± 0.68[ab]**	**7.7 ± 0.42[b]**	7.8 ± 0.39
Body depth at the anus	17.6 ± 0.70	16.8 ± 0.83	16.7 ± 0.82	16.7 ± 0.44
**Body width at the anus**	**13.3 ± 0.59[a]**	**12.0 ± 0.87[b]**	**12.6 ± 2.54[ab]**	12.0 ± 0.54
% of Head length				
**Head width**	**61.3 ± 2.57[a]**	**64.5 ± 2.56[b]**	**68.2 ± 4.44[c]**	62.4 ± 5.30
Head depth	68.8 ± 1.79	71.0 ± 2.40	68.9 ± 2.93	66.5 ± 2.71
Snout length	37.0 ± 2.71	35.7 ± 2.25	36.5 ± 1.04	37.1 ± 3.16
**Eye diameter**	**20.2 ± 1.17[a]**	**21.9 ± 1.42[b]**	**18.5 ± 0.81[c]**	20.6 ± 1.98
Jaw length	31.7 ± 2.16	33.4 ± 2.04	31.5 ± 2.81	31.9 ± 1.33

Number of fish used is 10 for each site except F3 where only 4 fish were used. Statistical comparisons were applied only to three sites because of the low number of fish in F3. Data in the same row with different alphabetical letters are significantly different (see text). The data for the first dorsal fin were compared only for males because of the sexual dimorphism[1]. The values in parentheses are for females. There were no females in the sample from F4. Mean ± SD.

Table S4. Summary of meristic analysis of *Periophthalmodon septemradiatus* collected from four sites along the Hau River

	F1-c	F5	F4	F3
Fin ray number				
First dorsal fin	14.4 ± 0.53 (5,5,6)	15.0 ± 0.87 (12)	13.6 ± 1.77	13,13 (5,8)
**Second dorsal fin**	**12.2 ± 0.42[ab]**	**12.9 ± 0.57[a]**	**12.1 ± 0.88[b]**	12.0 ± 0.00
Anal fin	10.0 ± 0.00	10.0 ± 0.00	10.0 ± 0.00	10.0 ± 0.00
Pelvic fin	5.0 ± 0.00	5.0 ± 0.00	5.0 ± 0.00	5.0 ± 0.00
Left pectoral fin	14.3 ± 0.82	15.0 ± 0.82	14.6 ± 0.97	14.0 ± 0.00
**Caudal fin**	**19.5 ± 0.85[a]**	**18.7 ± 0.67[ab]**	**18.3 ± 1.06[b]**	19.5 ± 0.29
Scale number				
Longitudinal series	47.3 ± 0.82	47.1 ± 0.57	46.7 ± 0.48	47.8 ± 0.50
Transverse backwards	7.1 ± 0.32	6.8 ± 0.42	7.4 ± 0.84	7.3 ± 0.50
Transverse forwards	5.0 ± 0.00	5.1 ± 0.32	5.5 ± 0.53	5.0 ± 0.00
Predorsal midline	18.8 ± 1.14	18.2 ± 0.42	18.7 ± 0.48	18.0 ± 0.00

Number of fish used is 10 for each site except F3 where only 4 fish were used. Statistical comparisons were applied only to three sites because of the low number of fish in F3. Data in the same row with different alphabets were significantly different (see text). The data for the first dorsal fin were compared only for males because of the sexual dimorphism[1]. The values in parentheses are for females. There were no females in the sample from F4. Mean ± SD.

Table S5. The locations of burrow density determination

Site ID	District, Province	Date	Tributary/Main channel	Latitude	Longitude
B1	Cu Lao Dung,	June 12, 2018	T	9°39'33.6"N – 9°39'41.0"N	106°09'21.5" E– 106°09'53.9"E
	Soc Trang		M	9°38'42.2"N – 9°38'28.4"N	106°08'23.3" E– 106°08'34.8"E
B2	Binh Thuy,	June 14–16, 2018	T	10°02'50.0"N – 10°02'50.8"N	105°43'22.2"E – 105°43'23.8"E
	Can Tho		M	10°02'51.9"N – 10°04'25.4"N	105°45'23.6"E – 105°47'31.9"E
B3	Thot Not,	June 17–18, 2018	T	10°09'26.7"N – 10°13'00.1"N	105°33'07.0"E – 105°38'39.0"E
	Can Tho		M	10°09'45.3"N – 10°09'49.3"N	105°38'30.6"E – 105°38'34.4"E
B4	Chau Thanh,	June 19–20, 2018	T	10°26'46.9"N –10°26'57.1"N	105°22'17.6" E– 105°22'21.1"E
	An Giang		M	10°27'1.8"N – 10°27'28.4"N	105°20'43.9" E– 105°22'5.2"E

Table S6. Cation concentrations of the main channel, tributary and burrow water in the upper reaches of the Hau River (B2-4) and in the estuarine island (B1)

	Upper reaches			Estuarine island	
	Main channel (N = 21)	Tributary (N = 15)	Burrow (N = 17)	Tributary (N = 5)	Burrow (N = 5)
Na^+	**0.45 ± 0.03^{a}**	**0.51 ± 0.07^{ab}**	**0.64 ± 0.32^{b}**	23.0 ± 1.8	22.8 ± 1.9
K^+	**0.16 ± 0.01^{a}**	**0.16 ± 0.02^{a}**	**0.26 ± 0.15^{b}**	2.12 ± 0.16	1.94 ± 0.23
Ca^{2+}	**0.57 ± 0.03^{a}**	**0.58 ± 0.03^{a}**	**0.82 ± 0.37^{b}**	1.50 ± 0.12	1.44 ± 0.22
Mg^{2+}	**0.25 ± 0.01^{a}**	**0.26 ± 0.02^{a}**	**0.42 ± 0.25^{b}**	3.47 ± 0.27	3.18 ± 0.39
Sr^{2+}	1.23 ± 0.09	1.23 ± 0.10	1.52 ± 0.58*	7.03 ± 0.04	6.49 ± 0.60

Values are in mmol l^{-1} except Sr (µmol l^{-1}). Mean ± SD. Values of the same ion with different superscripts are significantly different (Mann-Whitney test). No statistical test was applied to the data from the estuarine island, Cu Lao Dung (B1 in Fig. 1) due to the small sample size. The values for upper reaches are based on combined data from B2, B3 and B4 (see Fig. 1, same as the locations of burrow density determination given in Supplementary Table S5). *N = 16.

Table S7. Cytochrome *c* oxidase subunit II (COII) and D-loop (DL) molecular diversity indices for *Periophthalmodon septemradiatus*

Site ID	COII *n*	*h*	H*d*	H*r*	*π*	DL *n*	*h*	H*d*	H*r*	*π*
F1-a	10	5	0.667 ± 0.163	1.595	0.00242 ± 0.00073	10	7	0.911 ± 0.077	2.500	0.00381 ± 0.00061
F1-b	4	3	0.833 ± 0.222	2.000	0.00368 ± 0.00105	4	4	1.000 ± 0.177	3.000	0.00443 ± 0.00112
F1-c	10	7	0.867 ± 0.107	2.329	0.00331 ± 0.00076	10	7	0.867 ± 0.107	2.329	0.00413 ± 0.00073
F2	5	4	0.900 ± 0.161	2.400	0.00265 ± 0.00084	5	5	1.000 ± 0.126	3.000	0.00507 ± 0.00107
F3	41	12	0.802 ± 0.047	2.028	0.00240 ± 0.00029	41	27	0.954 ± 0.020	2.740	0.00502 ± 0.00038
F4	5	4	0.900 ± 0.161	2.400	0.00412 ± 0.00124	5	5	1.000 ± 0.126	3.000	0.00507 ± 0.00123
F5	13	7	0.833 ± 0.086	2.147	0.00211 ± 0.00044	13	11	0.974 ± 0.039	2.846	0.00551 ± 0.00063
Total	88	23	0.796 ± 0.036		0.00258 ± 0.00023	88	47	0.940 ± 0.017		0.00471 ± 0.00025

Sampling site ID, number of specimens (*n*), haplotype number (*h*), haplotype diversity (H*d*), haplotype richness (rarefied allelic richness[2]) (H*r*), and nucleotide diversity (*π*). H*d* and *π* are expressed as mean ± SD.

Table S8. Pairwise ϕst statistics for *Periophthalmodon septemradiatus* cytochrome *c* oxidase subunit II (COII) and D-loop (DL)

	F1-a	F1-b	F1-c	F2	F3	F4	F5
F1-a	-	-0.08527	-0.04575	-0.05719	-0.00246	-0.08156	0.00279
F1-b	0.08232	-	-0.05572	-0.05263	-0.02163	-0.11111	-0.06727
F1-c	-0.05023	-0.00317	-	-0.02967	0.00378	-0.02967	0.01762
F2	-0.04348	0.03481	-0.07547	-	-0.03367	-0.08696	-0.01687
F3	-0.02021	0.04661	-0.04101	-0.03551	-	-0.01236	0.00483
F4	-0.04348	-0.08541	-0.07547	-0.07143	-0.02278	-	-0.01687
F5	-0.01811	0.07801	-0.04206	-0.1006	-0.01767	-0.01937	-

ϕst values for COII below diagonal, ϕst values for DL above diagonal. There were no significant differences for any of the values ($p > 0.05$).

Table S9. Summary of field trips in the lower Mekong River in Vietnam

Year	Date	Lunar age	River surveyed
2015	Dec. 14–15	3.1–4.1	TC
2016	June 6–8	1.4–3.4	H, TC
	Sept. 29–30	28.1–29.1	H, TC
	Nov. 27–Dec.8	28.8–9.0	H
2017	April 10–16	13.4–19.4	H
	June 25–July 6	1.4–12.4	H
	Sept. 19–Oct. 10	28.7–20.3	H
	Dec. 12–27	24.0–9.2	H, TC
2018	Jan. 30	13.4	TC
	June 10–22	26.0–8.7	H

H, Hau River; TC, Tien River–Co Chien River

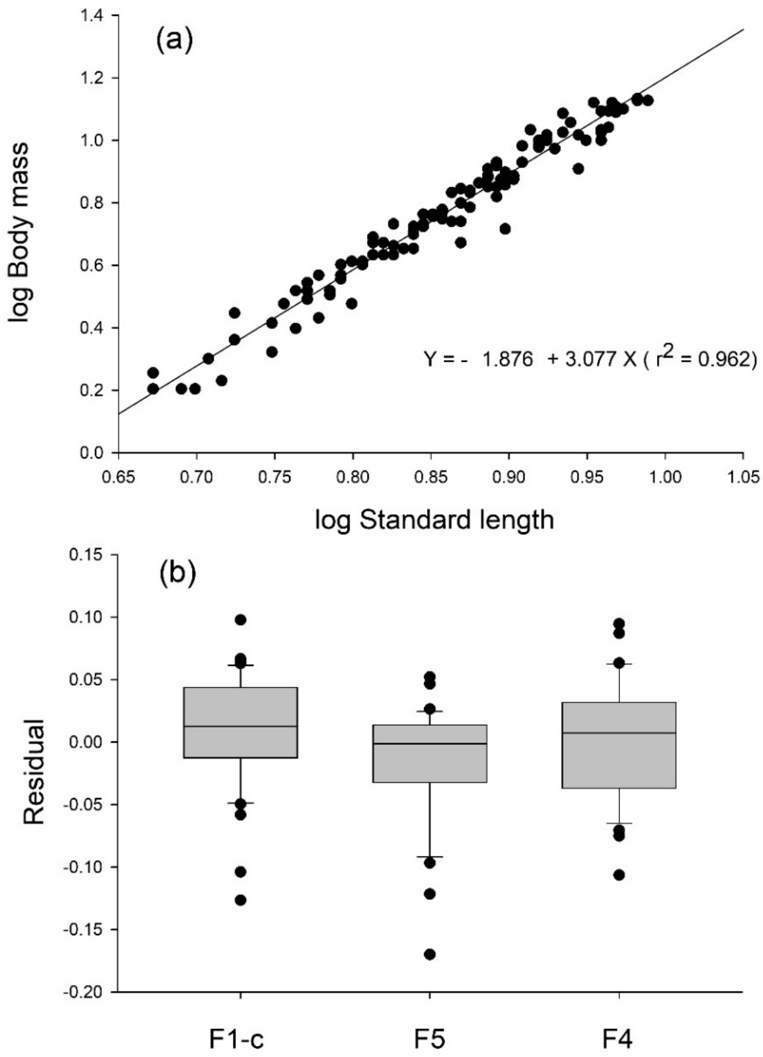

Fig. S1. (a) Relationship between body mass (g) and standard length (cm) of *Periophthalmodon septemradiatus* collected at F1-c, F4 and F5. (b) A box plot of the residuals from the regression shown in (a) for samples collected at F1-c, F4 and F5. The lines within the boxes mark the medians. The whiskers above and below the boxes indicate the 90th and 10th percentiles.

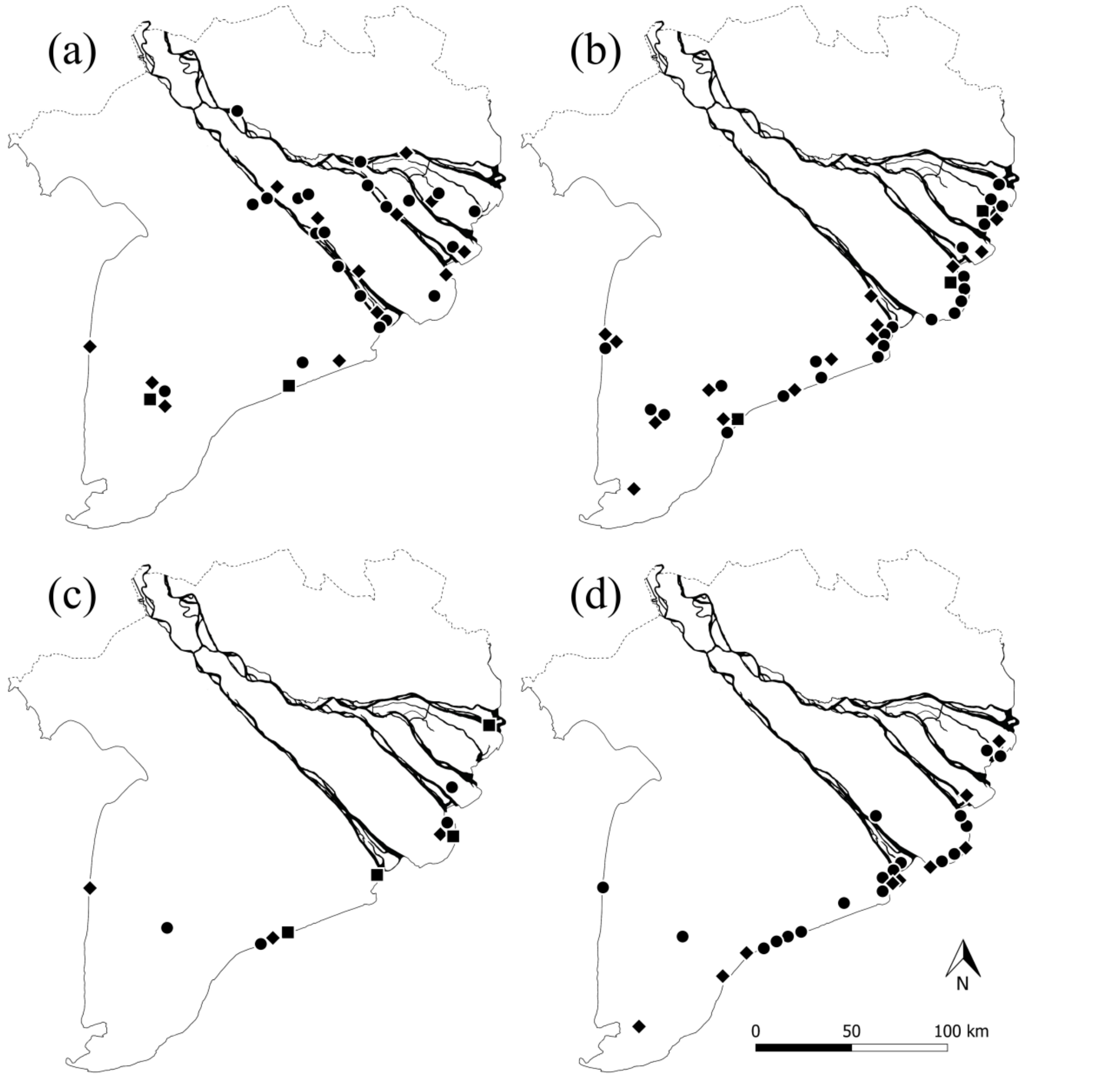

Fig. S2. The distribution of 11 species of oxudercine gobies in the Mekong Delta. (a) Filled circles, *Periophthalmodon septemradiatus*; filled squares, *Periophthalmodon schlosseri*; filled diamonds, *Parapocryptes serperaster*. (b) Filled circles, *Periophthalmus gracilis*; filled squares, *Periophthalmus variabilis*; filled diamonds, *Periophthalmus chrysospilos*. (c) Filled circles, *Apocryptodon madurensis*; filled squares, *Oxuderces nexipinnis*; filled diamonds, *Scartelaos histophorus*. (d) Filled circles, *Boleophthalmus boddarti*; filled squares, *Pseudapocryptes elongatus*. Data are from Kano et al[3]. The map was created with Qgis 3.4 (http://qgis.org/downloads/QGIS-OSGeo4W-3.4.7-1-Setup-x86_64.exe)[4] and Microsoft Powerpoint - version 1904 (https://products.office.com/en-ie/powerpoint) for labels and icons.

References

1. Murdy, E. O. A taxonomic revision and cladistic analysis of the oxudercine gobies (Gobiidae: Oxudercinae). *Rec Aust Mus Suppl* **11**, 1-93 (1989).

2. Librado, P. & Rozas, J. DnaSP v5: A software for comprehensive analysis of DNA polymorphism data. *Bioinformatics* **25**, 1451-1452 (2009).

3. Kano, Y. *et al.* An online database on freshwater fish diversity and distribution in Mainland Southeast Asia. *Ichthyol Res* **60**, 293-295, doi:10.1007/s10228-013-0349-8 (2013).

4. QGIS Development Team. QGIS Geographic Information System. Open Source Geospatial Foundation Project. http://qgis.osgeo.org (2018).
